# Understanding the phyllosphere microbiome assemblage in grape species (*Vitaceae*) with amplicon sequence data structures

**DOI:** 10.1038/s41598-019-50839-0

**Published:** 2019-10-04

**Authors:** Prashant Singh, Sylvain Santoni, Audrey Weber, Patrice This, Jean-Pierre Péros

**Affiliations:** AGAP, University of Montpellier, INRA-SupAgro, CIRAD, Montpellier, France

**Keywords:** Microbiology, Microbial ecology

## Abstract

Impacts of plant genotype on microbial assemblage in the phyllosphere (above-ground parts of plants, which predominantly consists of the set of photosynthetic leaves) of *Vitis vinifera* cultivars have been studied previously but the impact of grape species (under the grape family *Vitaceae*) was never investigated. Considering the fact, that the phyllosphere microbiome may have profound effects on host plant health and its performance traits, studying the impact of grape species in microbial taxa structuring in the phyllosphere could be of crucial importance. We performed 16S and ITS profiling (for bacteria and fungi respectively) to access genus level characterization of the microflora present in the leaf phyllosphere of five species within this plant family, sampled in two successive years from the repository situated in the Mediterranean. We also performed α and β-diversity analyses with robust statistical estimates to test the impacts of grape species and growing year, over a two-year period. Our results indicated the presence of complex microbial diversity and assemblages in the phyllosphere with a significant effect of both factors (grape species and growing year), the latter effect is being more pronounced. We also compared separate normalization methods for high-throughput microbiome data-sets followed by differential taxa abundance analyses. The results suggested the predominance of a particular normalization method over others. This also indicated the need for more robust normalization methods to study the differential taxa abundance among groups in microbiome research.

## Introduction

Plant phyllosphere is the niche of a disparate group of microbial communities of prokaryotes, eukaryotes, and viruses that interact with each other and with their host plant^1^. Due to the limited nutrient availability and fluctuating climatic conditions, phyllosphere is a dynamic and stressful habitat for its microbial colonizers^2,3^. The knowledge of these colonizers and drivers that might affect their assemblage might explain the mechanisms that control operations at the intersection between plants, microbiome, and the atmosphere. However, phyllosphere has been largely overlooked in most of the grape related microbiome studies and researches were mainly focused on rhizosphere (root and soil colonizers) and endosphere (inside tissue colonizers)^4–9^. Most of these studies including few in phyllosphere^10–12^ analyzed the main drivers for microbial assemblage on *Vitis vinifera*, the most widely cultivated species for wine and raisins. They suggested that the environmental conditions at different geographic locations (or terroir) and growing seasons are important drivers in the rhizosphere, endosphere or phyllosphere^10,11^. Cultivars or genotype interaction with the environment also seemed to play a role in this assemblage^10,12^. Since most of the current breeding schemes in grapevines involve other *Vitis* species as pest resistance sources, we wanted to inquire about the effect of the plant species on microbial assemblage. Furthermore, as the selection of microbiome could lead to new plant breeding strategies of next-generation, identifying microbiome differences among grape species could be an interesting opportunity for new grape breeding schemes to develop resistant, healthy and more productive varieties^13^. Moreover, grape associated epiphytes have been recently established as promising biocontrol agents (BCA) against fungal pathogens^14^ of *Vitis vinifera*, the species level variation could offer us hints of new potential players for BCA.

In this work, we sampled epiphytes from five different species in the *Vitaceae*^15,16^ to find major microbial colonizers in their phyllosphere and compared the impact of grape species and growing year on the phyllosphere microbiome assemblage. To mitigate the confounding effect of environmental conditions at different geographic locations (or terroir), we sampled all five grape species (*Vitis vinifera cv*. *Cabernet-Sauvignon*, *Vitis pentagona, Vitis riparia, Muscadinia rotundifolia*, and *Parthenocissus quinquefolia*) from Montpellier-SupAgro repository of INRA in the Mediterranean. These species were chosen to study the genotype effect at species level within subgenus *Vitis*, at the subgenus level (subg. *Vitis* versus subg. *Muscadinia*) and at the genus level (*Vitis* versus *Parthenocissus*). The three species within subg. *Vitis* are genetically distant^15,17^ and cover the distribution range of this subgenus in the Northern Hemisphere with the European *V. vinifera*, the Asian *V. pentagona* and the American *V. riparia*^16,18,19^. If the genotype is a major driver of the phyllosphere microbiome assemblage, we, therefore, expected increasing differences in microbial assemblages according to the genetic distance between plant species.

From a statistical point of view, we compared the differential abundance of microbial genera using three separate data normalization methods to predict the better normalization approach for grape related or other microbiome data. Normalization is the data transformation process enabling a true comparison of statistics from separate measurements by discarding artefactual biases from the original measurements. Most of the microbiome studies including grapevines^9,20^ still use the most standard statistical methods for differential abundance analysis without testing the data distribution and transformation. In microbial ecology, rarefying samples to even sequencing depth is standard normalization method but is also not an ideal one, as it potentially reduces statistical power relying upon how much data is filtered and does not address the challenge of compositional data^21^. The log ratio transformation method is also widely accepted by researchers and statisticians and in various high dimensional studies^22,23^. Here, we used few recently published data transformation methods of cumulative sum scaling (CSS)^24^, DESeq2^25^ and log ratio to normalize our zero-inflated taxa abundance data and compared estimates of differentially abundant genera between two growing years and grape species.

## Results

### Phyllosphere exhibits diverse bacterial and fungal communities

**Figure 1:**
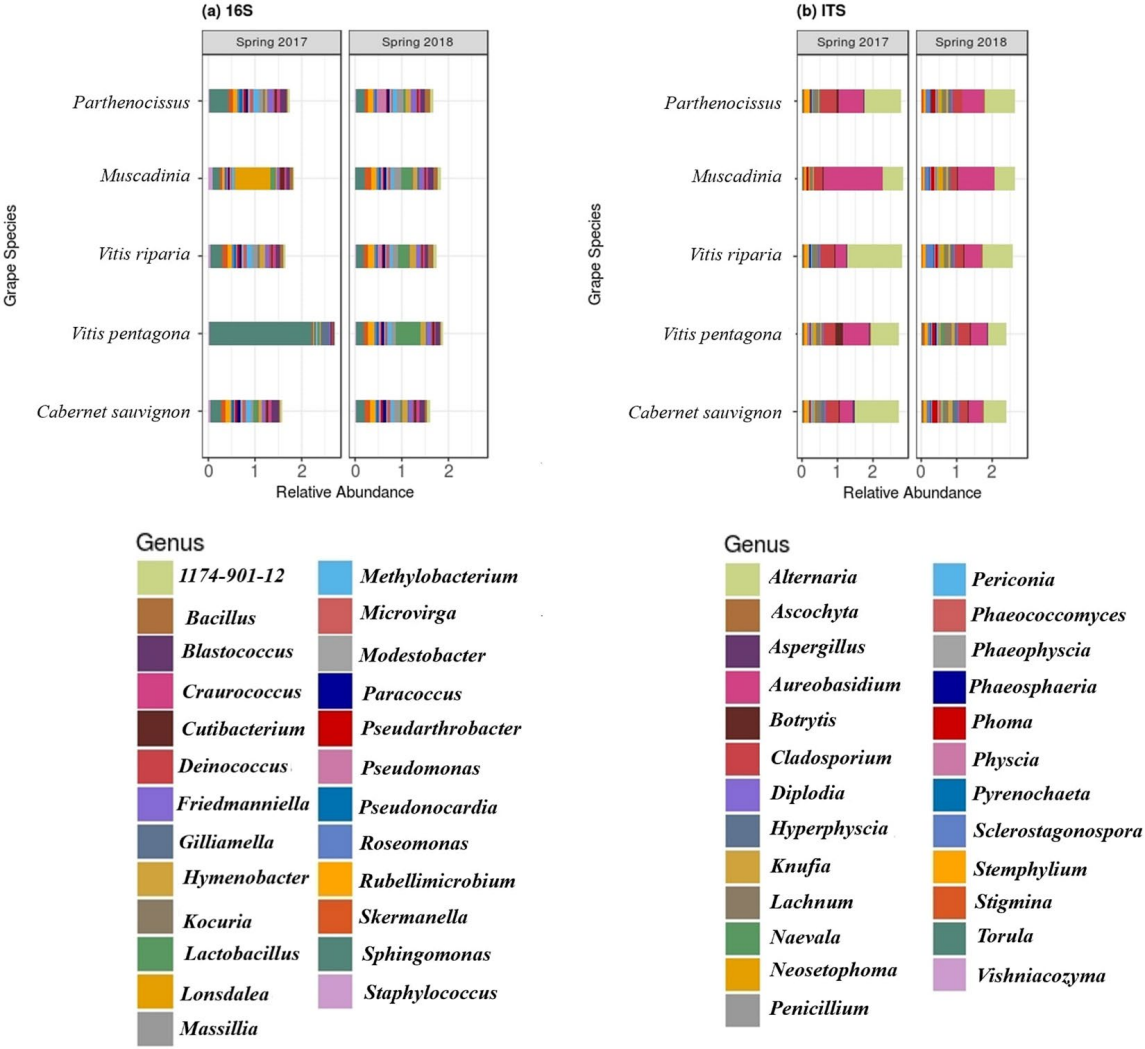
Mean (**a**) bacterial and (**b**) fungal relative abundance of top 25 genus present across samples i.e leaf phyllosphere of five grape species.

Millions of amplicon reads were processed from both data-sets (16S and ITS) and 10825 bacterial and 5252 fungal amplicon sequence variants (or operational taxonomic units-OTUs) were obtained (Table 1). After assigning the OTUs to phylum level more than 73% bacterial OTUs and ~95% of fungal OTUs were assigned to phylum level. Unknown sequences corresponded to ~27% and ~5% for bacterial and fungal data respectively, meaning that the taxonomic assignment procedure was not able to assign any microorganism to these sequences. *Proteobacteria* (relative abundance ~15%) and *Cyanobacteria* (~14.8%) were the most dominated phyla across the samples followed by *Firmicutes* (~3%) and *Actinobacteria* (~1.3%). On the other hand, samples were heavily dominated by fungal phyla of *Ascomycota* (~91%) followed by *Basidiomycota* (~9%). After gloming of these OTUs at the genus level, 677 bacterial and 434 fungal genera were recovered. Out of these, *Sphingomonas, Methylobacterium, Rubelimicrobium, Blastococcus* and *Alternaria, Aureobasidium, Cladosporium, Lachnum* were most abundant bacterial and fungal genera, respectively (Fig. 1).

**Table 1:** The total number of reads at each step of bacterial microbiome data processing.

Number of samples	Reads in	Reads out (Filtered)	Denoised	Chimera removal	OTUs
**16S Data**					
30	5,568,565	4,538,503	4,139,738	3,9763,42	10,825
**ITS Data**					
30	2,7429,06	2,674,944	2,411,474	2,407,048	5,252

### Microbial communities clustered distinctly with year and grape species

**Figure 2:**
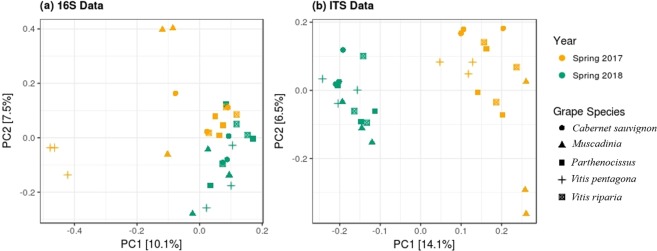
PCoA ordinations of (**a**) bacterial and (**b**) fungal communities derived from leaf phyllosphere at two growing years, using Bray-Curtis distance matrix. Both the axis explains ~20% of variations. The shape represents grape species (N = 30).

**Figure 3:**
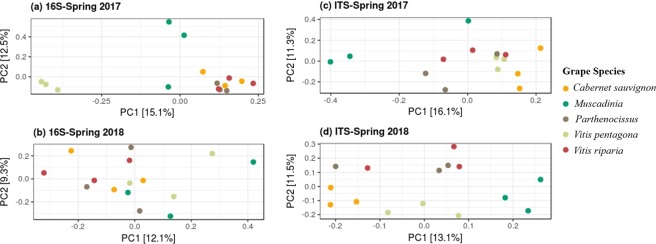
PCoA ordinations of bacterial (**a**,**b**) and fungal (**c**,**d**) communities derived from leaf phyllosphere at spring 2017 and spring 2018 separately, using Bray-Curtis distance matrix.

Multidimensional scaling (or principal coordinate analysis; PCoA) on microbial abundance data showed that the samples from each year clustered together and were distinct from each other (more predominant in ITS data, Fig. 2), confirmed the significant impact of the growing year on microbial community structuring in the phyllosphere. Clustering among grape species was not prominent (Fig. 2) but performing PCoA on the subset of the data (i.e. on separate data for each year) displayed a lower but significant impact of grape species in shaping phyllosphere microbiome, especially the fungal microbiome (Fig. 3). Permutational analysis of variance (PERMANOVA) statistics according to Year, Species and the interaction term (Year × Species), further confirmed the hypothesis (Table 2) that the synergy between the environment and the plant genotype could be held responsible for microbial community structuring in the phyllosphere.

**Table 2:** Components predicting the impacts of Year and Grape Species on the leaf microbiome.

Data	ANOVA (on α-diversity measures)	PERMANOVA (on PCoA clusters)
**16S**		
Year	at F = 5.725, P = 0.0076**	R^2^ = 0.269, F = 1.811, P = 0.0001***
**Year × Grape Species**	**at F** = **9.022, P** = 0.00138**	**R**^**2**^ = **0.154, F** = **1.737, P** = 0.0001***
Grape Species (Spring 2017)	at F = 3.752, P = 0.041	R^2^ = 0.379, F = 1.525, P = 0.0001***
Grape Species (Spring 2018)	at F = 1.743, P = 0.217	R^2^ = 0.304, F = 1.134, P = 0.031*
**ITS**		
Year	at F = 49.261, P = 1.24e-07	R^2^ = 0.101, F = 3.532, P = 0.0001***
**Year** × **Grape Species**	**at F** = **57.340, P** = **2.71e-07**	**R**^**2**^ = **0.112, F** = **3.767, P** = 0.0001***
Grape Species (Spring 2017)	at F = 2.843, P = 0.08	R^2^ = 0.325, F = 1.206, P = 0.0038**
Grape Species (Spring 2018)	at F = 1.274, P = 0.34	R^2^ = 0.334, F = 1.257, P = 0.0001***

**Figure 4:**
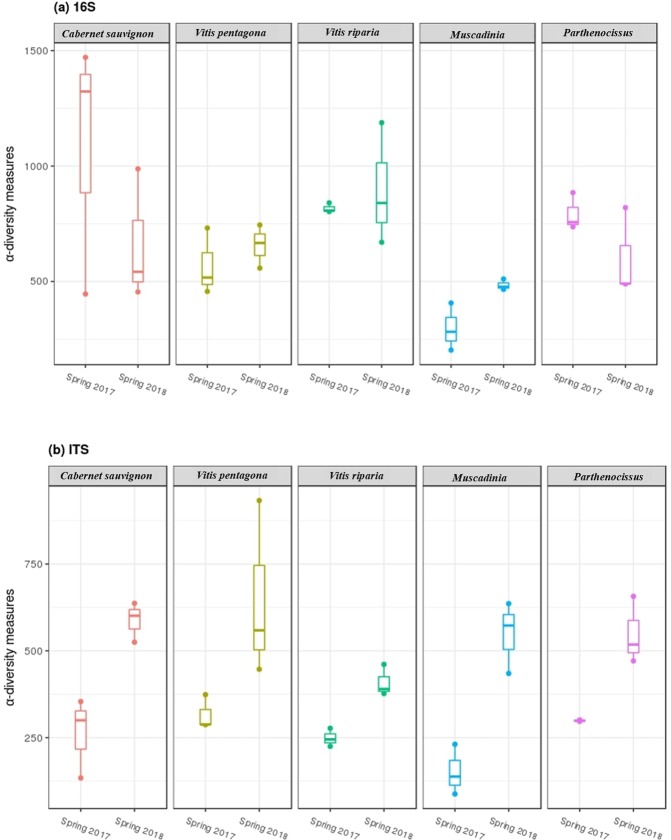
Box plots of mean *observed α-diversity* measures or unique OTU-richness between Spring of 2017 and 2018 for (**a**) bacterial and (**b**) fungal data. For each box plot, the top point is maximum observation, the lower point is minimum observation, top of the box is the third quartile, the bottom of the box is the first quartile, the middle bar is median values and color represent grape species (N = 30).

Furthermore, *observed α-diversity* estimates of bacterial and fungal OTUs of each grape species (within each growing year, Fig. 4) revealed that the unique OTU-richness in the phyllosphere of each grape species significantly differed between the two years (Table 2). This reconfirmed the major impact of the growing year in shaping phyllosphere microbial assemblage. Although OTU-richness estimates did not vary according to grape species (P~0.05), the interaction term (Year × Species) displayed strong differences in the richness of unique OTUs (Table 2).

### Comparing normalization methods

**Figure 5:**
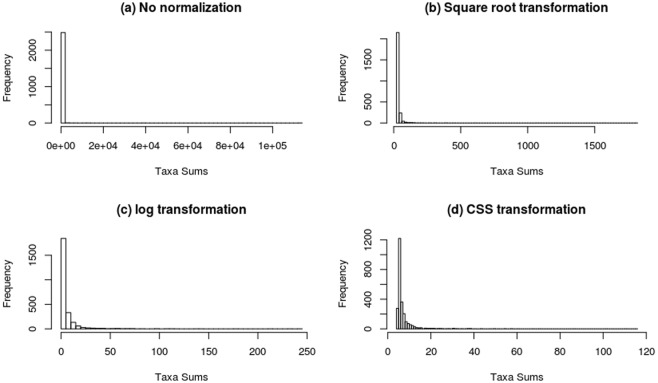
Histograms of the distribution of taxa sums across samples using (**a**) no normalization (**b**) Square root transformation (**c**) log transformation and (**d**) CSS transformations.

Most of the microbiome data doesn’t follow a normal distribution for taxa abundance across the samples^9,20,25^ and our data-sets were not the exceptions. Using three separate data transformation methods (square root, log-ratio, and CSS) we were unable to achieve proper normalization of our data-sets, and even after transformation, most of the distribution followed negative binomial distribution except CSS which performed better than others (Fig. 5). We used these transformed data for differential abundance analysis of each taxon (at genus level) according to the grape species and growing year (DESeq2 was applied to square root transformed data, to handle negative binomial distribution).

**Figure 6:**
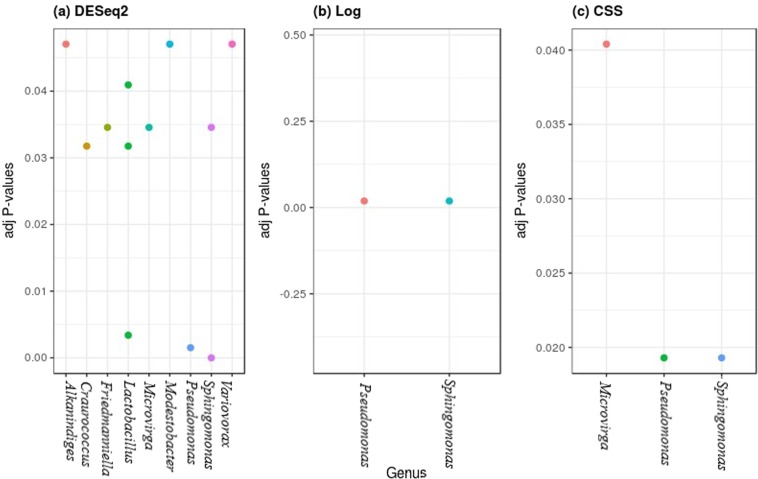
Separate normalization methods identified different bacterial taxa that significantly contributed (adj P < 0.05) to differences between two growing years.

**Figure 7:**
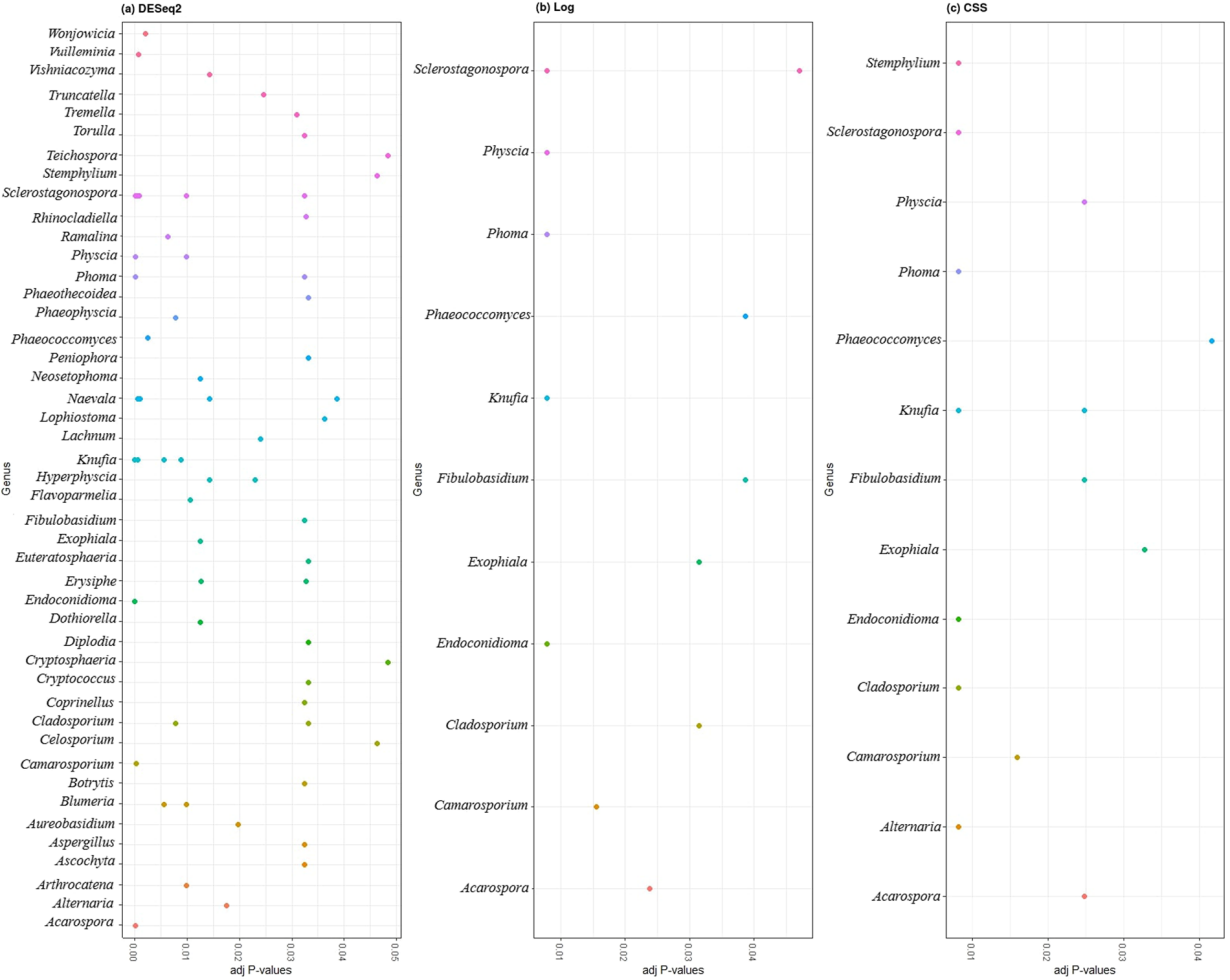
Separate normalization methods identified different fungal taxa that significantly contributed (adj P < 0.05) to differences between two growing years.

Performing multiple testing with FDR corrected P-values (adj-P value < 0.05) gave nine bacterial genera for DESeq2 as compared to two and three genera for log and CSS transformed data between the two years. Similarly, 45 fungal genera were obtained for DESeq2 as compared to 11 and 13 genera for other methods between the two years (Figs 6 and 7). The same testing was performed on data-sets of Spring 2017 and Spring 2018 separately to identify differential taxa abundance among five grape species and the results were similar (Tables 3 and 4). DESeq2 gave a higher number of genera as compared to other methods (probably an overestimation). Combining normalization performance on taxa abundance across samples and identification of differentially abundant genera, indicated that CSS normalization method worked better and was statistically more robust, i.e. fewer false positives and lower false discovery rates, as compared to other methods.

**Table 3:** Differential taxa abundance (bacterial taxa, genus level) among Grape Species at each year.

Method	Genus	Adj P-value	FDR
**Spring 2017**			
CSS	*Cutibacterium*	0.033	0.001
	*Rubelimicrobium*	0.0171	0.001
	*Sphingomonas*	0.034	0.018
Log	*Cutibacterium*	0.038	0.017
	*Rubelimicrobium*	0.168	0.001
DESeq2	*Cutibacterium*	0.038	0.128
	*Rubelimicrobium*	0.0192	0.212
	*Sphingomonas*	0.042	0.201
	*Microvirga*	0.022	0.113
	*Rhodococcus*	0.022	0.113
**Spring 2018**			
CSS	*Hymenobacter*	0.036	0.061
	*Rubelimicrobium*	0.036	0.061
Log	*Hymenobacter*	0.034	0.045
	*Rubelimicrobium*	0.034	0.045
	*Methylobacterium*	0.039	0.045
DESeq2	*Hymenobacter*	0.027	0.118
	*Rubelimicrobium*	0.027	0.118
	*Paracoccus*	0.027	0.118
	*Methylobacterium*	0.038	0.221
	*Pedobacter*	0.038	0.221
	*Kineococcus*	0.038	0.221
	*Spirosoma*	0.043	0.312
	*Blastococcus*	0.043	0.312

**Table 4:** Differential fungal taxa abundance (fungal taxa, genus level) among Grape Species at each year.

Method	Genus	Adj P-value	FDR
**Spring 2017**			
CSS	*Aspergillus*	0.0011	0.022
	*Alternaria*	0.0171	0.033
Log	*Aspergillus*	0.0012	0.017
	*Alternaria*	0.0012	0.017
	*Botrytis*	0.0131	0.112
DESeq2	*Hyphodontia*	0.006	0.081
	*Aspergillus*	0.026	0.119
	*Alternaria*	0.026	0.119
	*Botrytis*	0.026	0.119
**Spring 2018**			
CSS	*Alternaria*	0.024	0.038
	*Sclerostagonospora*	0.024	0.038
	*Truncatella*	0.032	0.048
Log	*Alternaria*	0.027	0.0199
	*Sclerostagonospora*	0.027	0.0199
	*Truncatella*	0.034	0.0231
DESeq2	*Knulfa*	0.021	0.132
	*Alternaria*	0.021	0.132
	*Sclerostagonospora*	0.021	0.132
	*Truncatella*	0.037	0.172
	*Botrytis*	0.037	0.172

## Discussion

Microbial colonizers of endosphere and rhizosphere associated with *Vitis vinifera* cultivars (or grapevines) have been studied before quite extensively^4,8,9^. Recently, grapevine’s phyllosphere has also been explored by researchers^10–12^, but these habitats were not explored in the context of different grape species of *Vitaceae* prior to this study. At first, we explored the phyllosphere of five different grape species using the culture-independent methods of 16S and ITS profiling. Out of the complex microbial diversity, *Sphingomonas* and *Methylobacterium* were the most dominant bacterial genera. The fungal community was dominated by *Aureobasidium, Cladosporium* and *Alternaria*. These results are in line with the previous phyllosphere related works^26–28^, which suggests that the phyllosphere is usually dominated by these genera because of their important functions (e.g- plant growth, plant defense, and biocontrol agent, etc.)^11,29^. For example, phyllosphere is usually quite rich in *Methylobacterium*, which can benefit the plant by absorbing the methanol (gaseous) released by the stomata and produces a growth promoting agent for the seedlings of some crop plant^29^. Biocontrol potential of some of these genera (e.g- *Sphingomonas* and *Aureobasidium*) have been reported previously^30–34^ and should be explored in future studies in the context of grapes and in collaboration with other taxa with major or minor abundances. As the microbial communities in the phyllosphere can modulate leaf susceptibility to infections, it can protect the plant from foliar diseases^30,35^. Hence, the microflora of the phyllosphere and the local environmental conditions can thus regulate the fitness of their host plant. The current major challenge is to learn the properties of these microbial communities in the phyllosphere (e.g- taxonomic and functional diversity and microbial network structure) that will be beneficial under changing climatic conditions to cultivate properties of biocontrol and robustness in grape plants to sustain the productivity, yield, and resilience of agricultural systems. Among the diverse and complex communities found in the phyllosphere the genus, *Massilia* is also noteworthy as its presence was quite consistent during the two years. It is a major pollutant of an aerosol with agricultural applications^36,37^ and could be an indicator of agricultural practices in the Mediterranean. The genus *Rubelimicrobium* was also one of the major and dominant genera found as a leaf epiphyte. Few species of this genus were isolated from soil^38^, and this could be a shred of evidence to support the claim that the soil microbiota may influence the epiphyte compositions^9^.

Secondly, we assessed the relative impacts of grape species and the growing year to shape the assemblage of the microbial communities in the phyllosphere. Morphological structures of leaves, its chemistry and physiology may differ according to the grape species as all these traits can be impacted by plant genetics, and this variation may also lead to different blends of microbial community structures^39,40^ in the phyllosphere. In previous studies, minor impacts of grapevine genotypes (at cultivar level) have been found^10,12^ and we expected greater impacts of genotypes once we sample from different species in order to increase genetic distances between them but our analysis indicated that the growing year had much stronger impacts than grape species in microbial community structuring. At each individual growing year, grape species also showed some influence in shaping this assemblage (especially for fungal assemblage) but that is noticeably weaker in comparison with the growing year. Statistical estimates of α and β-diversity suggest that the plausible hypothesis could be the species-climate interaction that is responsible for recruiting microbes on the leaf surface of different grape species. This interaction could be multilayered as slight changes in meteoclimatic parameters (daylight exposure, increased atmospheric CO_2_ concentrations, and temperatures) between two growing years may alter certain important soil characteristics (moisture volume, amount of root exudates, soil temperature etc.), which in turn may induce major changes in soil microbiome and ultimately in epiphytic communities^9,41,42^.

Lastly, we also analyzed the impacts of the data normalization methods to detect the differential taxa abundance among different groups. Microbiome datasets like taxonomy reads or OTU counts from amplicon sequencing experiments or differential expression (RNA-Seq) data are often scattered and have frequent zeros. In order to fit these overdispersed microbiome datasets, negative binomial (NB) is often applied^24,43,44^. For example, an NB model was fitted in several microbiome studies related to Parkinson’s disease and to analyze the effect of edible cricket consumption on gut microbiome^45–47^. Similarly, an NB model was also used for identifying differential sequence tag abundance and for detecting differentially abundant features in clinical metagenomic samples^44,48^. Zero-inflated Gaussian (ZIG) mixture model was proposed^24^ in 2013, which uses a new cumulative sum scaling (CSS) normalization technique to correct the bias in measuring the differential abundance introduced by total sum normalization. This model precisely evaluates the probability that an observed zero is generated due to the undersampling or from the count distribution (absence of the taxonomic feature in the samples). We evaluated one NB method (DESeq2), CSS method and log transformation of the data to generate normalized counts and performed multiple testing on differential taxa abundance. Our results suggested that the CSS method worked finer than other methods in obtaining the normalized counts and gave statistically more robust estimates of differential taxa abundance.

## Conclusion

Our study revealed the existence of heterogeneous microbiome in the leaf phyllosphere of *Vitaceae* and we speculated that the multilayer species-climate interaction could be the reason for the microbiome assemblage in the phyllosphere. We have also inferred the advantages of CSS normalization over other methods, however, this method needs to be further evaluated with the sufficient amount of other microbiome studies.

## Material and Methods

### Sampling, isolation of phyllosphere microbes and DNA extraction

Leaf samples (from each of the 5 grape species) were collected from the repository of the agronomic school SupAgro at Montpellier, France (Mediterranean). 18–20 fully developed asymptomatic leaves were randomly sampled from 3–4 plants of each grape species in the Spring season (mid of May 2017 and 2018, before fungicide spraying) to make three replicates. The sample washing procedure was adopted by our previously published protocol^12^ and was performed with isotonic sodium chloride solution (0.15 M) with 0.01% Tween 20 in 50 ml propylene tubes using a horizontal shaker at 100 RPM for 1hr^10^. To maximize the microbial recovery from the leaf surface, samples were given an ultrasonic bath for 7–10 minute using Ultrasonic Cleaner (Branson 5510). Afterward, the remaining solution was centrifuged at 4,000 g and microbial pellets were transferred in 2-ml Eppendorf tubes and stored at −20 °C. DNA was extracted from each sample using the ZymoBiomics DNA MicroPrep Kit (Zymo Research, USA).

### PCR amplification and amplicon sequence library preparation

The 16S ribosomal gene (V4 region) was amplified using 515 F and 806 R primers to characterize bacterial communities. Modified ITS9 and ITS4 primers targeting the ITS2 region were used to access fungal community diversity and abundances. Sequencing libraries were prepared using two-step PCR reactions. Amplification of the target regions and the addition of Illumina Nextera transposase sequence to the amplicons was performed in step one PCR (or PCR1). Both PCR1 primers (forward and reverse) were amended with frameshift (FS) sequences in their 5′ overhang to improve overall read quality and sequence diversity^49^. 25 μL reactions (with 30 ng of sample DNA) using the KAPA HiFi HotStart (KAPA Biosystems, USA) PCR mix (initial denaturing at 95 °C followed by 30 cycles of denaturing at 95 °C for 30 s, primer annealing at 57 °C for 60 s and primer extension at 68 °C for 60 s) were used to perform PCR1. Purification of amplicons was achieved using Agencourt AMPure XP beads (Beckman Coulter, USA) at a bead-to-DNA ratio of 0.7:1. Amplicons were then resuspended in 30 μL MilliQ water and evaluated in agarose gels. In PCR2 (Illumina dual indexing PCR), each cleaned PCR1 product within the same sample received a unique combination of forward and reverse primers (respectively, N7 and S5 Illumina dual index oligos). Samples were again cleaned afterward, using AmPure XP magnetic beads, pooled in equimolar concentrations and sequenced using 2 × 250 bp MiSeq v2 sequencing (Illumina Inc., San Diego, CA, USA). ZymoBIOMICS microbial community standard and DNA extraction buffer were used for this library preparation as a positive and negative control respectively following the manufacturer’s instructions.

### Data analysis

Paired-end sequence reads from 16S and ITS sequences were filtered, trimmed and processed with the *dada2* v1.8 (R Bioconductor package)^50^. The fastqPairedFilter function of the *dada2* was used to trim primers and discard bases with low-quality scores (q < 11). A core Divisive Amplicon Denoising Algorithm (DADA) was performed on these filtered files and amplicon sequence variants (or OTUs) were inferred^10,50^. Chimeras were removed afterward using the removeBimeraDenovo function of the same *dada2* package (Table 1 represents the total number of reads retained after performing each of these steps). Taxonomy was assigned to bacterial and fungal OTU sequences using the RDP classifier^51^ and UNITE data base^52^ respectively with k-mer size 8 and 100 bootstrap replicates.

Later, the *phyloseq* package^53^ was used to get the α and β-diversity estimates. The estimate_richness function of the *phyloseq* gave *observed α-diversity* measures within sample categories. Relative abundances of microbial genera were plotted using the *ggplot2* package^54^ after gloming the data at the genus level (using the tax_glom function of the *phyloseq*) and transforming genus abundance data into relative counts. PCoA ordination was performed on variance stabilized log-transformed data using the Bray-Curtis dissimilarity matrix and visualized by using their base functions in the *phyloseq* package.

### Data transformations and statistical analysis

Square root transformation of the data was performed on taxa counts using sqrt(1 + x) function. Log transformation was done using log(1 + x) function on taxa counts. DESeq2 normalization was performed using the phyloseq_to_deseq2 function of the *DESeq2* package^25^. CSS normalization was done using the *metagenomeSeq* package^24^. Multiple testing was performed using the mt function of the phyloseq package after each data transformations to identify differentially abundant taxa between groups.

All computational analyses were performed in R-environment v3.3.4 (R Core Team, 2017) and the statistical significance was assessed at P < 0.05 throughout, and we adjusted P-values for multiple comparisons (whenever necessary) according to the Benjamini and Hochberg method to control False Discovery Rate (FDR)^55^. We performed ANOVA (analysis of variance)^56^ among sample categories while measuring the observed estimates of α-diversity. To assess the statistical significance of PCoA clusters according to the sample categories, stratified permutational multivariate analysis of variance (PERMANOVA) with 9999 permutations was conducted (at α = 0.05) on all principal coordinates obtained during PCoA ordinations with the adonis command (with appropriate model matrix) of the *vegan* package^57^.

## Data Availability

Data is provided fully in the result section of this paper and the sequence data is uploaded at the institutional server of INRA and can be obtained upon reasonable request to authors.
